# Clinical Outcomes of 3D-Printed Bioresorbable Scaffolds for Bone Tissue Engineering—A Pilot Study on 126 Patients for Burrhole Covers in Subdural Hematoma

**DOI:** 10.3390/biomedicines10112702

**Published:** 2022-10-26

**Authors:** Emma M. S. Toh, Ashiley A. Thenpandiyan, Aaron S. C. Foo, John J. Y. Zhang, Mervyn J. R. Lim, Chun Peng Goh, Nivedh Dinesh, Srujana V. Vedicherla, Ming Yang, Kejia Teo, Tseng Tsai Yeo, Vincent D. W. Nga

**Affiliations:** 1 Yong Loo Lin School of Medicine, National University of Singapore, Singapore 117597, Singapore; emmatoh@u.nus.edu (E.M.S.T.); ashileyat@u.nus.edu (A.A.T.); 2 Division of Neurosurgery, Department of Surgery, National University Hospital, Singapore 119228, Singapore; e0366008@u.nus.edu (A.S.C.F.); zhangjohnjy@gmail.com (J.J.Y.Z.); mervynlim@u.nus.edu (M.J.R.L.); chun_peng_goh@nuhs.edu.sg (C.P.G.); nivedh.dinesh@gmail.com (N.D.); venkata.vedicherla@mohh.com.sg (S.V.V.); surteok@nus.edu.sg (K.T.); tseng_tsai_yeo@nuhs.edu.sg (T.T.Y.); 3 Division of Neurosurgery, Department of Surgery, Khoo Teck Puat Hospital, Singapore 768828, Singapore; yangming.yami@gmail.com

**Keywords:** chronic subdural hemorrhage, burrhole cover, cosmetic outcomes, polycaprolactone, bioresorbable

## Abstract

Burrhole craniostomy is commonly performed for subdural hematoma (SDH) evacuation, but residual scalp depressions are often cosmetically suboptimal for patients. Osteoplug^TM^, a bioresorbable polycaprolactone burrhole cover, was introduced by the National University Hospital, Singapore, in 2006 to cover these defects, allowing osseous integration and vascular ingrowth. However, the cosmetic and safety outcomes of Osteoplug^TM^-C—the latest (2017) iteration, with a chamfered hole for subdural drains—remain unexplored. Data were collected from a single institution from April 2017 to March 2021. Patient-reported aesthetic outcomes (Aesthetic Numeric Analog (ANA)) and quality of life (EQ-5D-3L including Visual Analog Scale (VAS)) were assessed via telephone interviews. Clinical outcomes included SDH recurrence, postoperative infections, and drain complications. Osteoplug^TM^-C patients had significantly higher satisfaction and quality of life compared to those without a burrhole cover (ANA: 9 [7, 9] vs. 7 [5, 8], *p* = 0.019; VAS: 85 [75, 90] vs. 70 [50, 80], *p* = 0.021), and the absence of a burrhole cover was associated with poorer aesthetic outcomes after multivariable adjustment (adjusted OR: 4.55, 95% CI: 1.09–22.68, *p* = 0.047). No significant differences in other clinical outcomes were observed between Osteoplug^TM^-C, Osteoplug^TM^, or no burrhole cover. Our pilot study supports Osteoplug^TM^-C and its material polycaprolactone as suitable adjuncts to burrhole craniostomy, improving cosmetic outcomes while achieving comparable safety outcomes.

## 1. Introduction

Three-dimensional (3D)-printed scaffolds are increasingly being used in medical bioengineering for reconstruction, tissue regeneration, and tissue repair [1]. As a production method, 3D printing allows biomaterials to be precisely controlled and easily produced, promoting attachment to surrounding tissues and organs while providing structural support [2]. This lends importance to fields such as plastic/reconstructive surgery and neurosurgery, where 3D-printed scaffold implants can be integrated with the surrounding bone to restore defects in areas such as maxillofacial reconstruction [3], cranioplasty [4] and, recently, for burrhole restoration after chronic subdural hematoma (cSDH) evacuation. Increasingly, implants of polycaprolactone (PCL)—a semicrystalline linear polymer—have become a favorable option for biomedical and commercial applications [5].

Various materials for burrhole covers have been proposed, including autologous bone grafts and dust [6,7], biomaterials such as porous polyethylene [8], poly(methyl methacrylate) (PMMA) [9], hydroxyapatite (HA) [10,11], mineralized collagen [12], and titanium [13]. These materials can potentially be toxic to cells [14], lack appropriate structures and interconnected porosity for vascular infiltration, or have limited biodegradability, introducing concerns for potential long-term complications. Finally, current burrhole cover designs limit drain placement. 

An original bioresorbable PCL burrhole cover named Osteoplug^TM^ was designed by a team from the National University Hospital, Singapore, along with the Department of Biomedical Engineering at the National University of Singapore in 2006 to provide a scaffold for tissue regeneration in skull defects [15]. Developed by Osteopore International Pte Ltd., this implant was subsequently patented and has since received US Food and Drug Administration (FDA) approval for human clinical use. The PCL material of Osteoplug^TM^ has previously demonstrated radiological and histopathological evidence for vascular ingrowth and osseous integration of the implant into surrounding calvarial bone, owing to its interconnected porous microstructure, and leaves no foreign material remaining in the long term [16,17]. 

**Figure 1:**
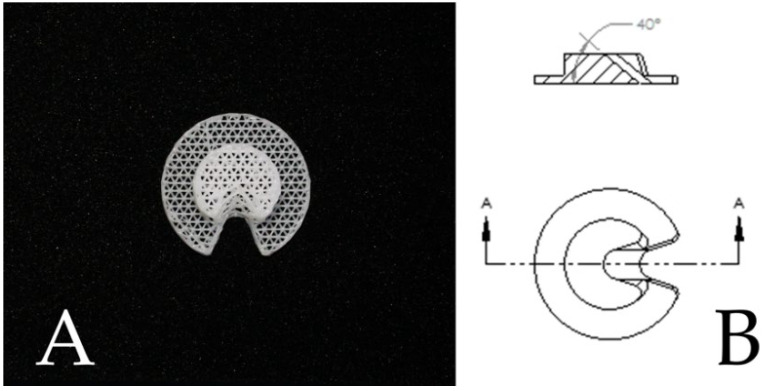
(**A**) A photograph of Design 2, which allows for placement of a subdural drain. (**B**) A drawing of Design 2 demonstrating the chamfered hole design at 40°.

The most recent iteration of Osteoplug^TM^ is Osteoplug^TM^-C. Introduced in 2017, it has been modified to include a chamfered hole at a 40-degree angle to allow for a drain to be easily passed through to the subdural space through its “C”-shaped opening, and its roof fits snugly into a burrhole without additional screws (Figure 1). This redesign is useful, as drains are increasingly used for cSDH surgery to safely reduce recurrence and improve outcomes [18]. The older versions of Osteoplug^TM^ have been described in our institution to have low infection rates [17] and low cSDH recurrence rates comparable to those found in other published literature [16]. However, no direct evaluation of the latest Osteoplug^TM^-C compared to patients without burrhole covers has been performed, and no objective data on cosmetic differences have been studied. The designs of older versions of Osteoplug^TM^ and Osteoplug^TM^-C are henceforth referred to as Design 1 and Design 2, respectively, as shown in Table 1. 

Burrhole craniostomy is a commonly used technique in neurosurgery for evacuation of cSDH. cSDH is defined as a collection of blood and blood breakdown products between the brain’s surface and the dura overlying it [19]. Diagnosis of this condition is made via computed tomography (CT) scan. In the procedure, a hole is drilled into the skull, approximately 14 mm × 14 mm in dimensions. A burrhole cover may or may not be placed to cover the defect as part of the surgical procedure.

**Figure 2:**
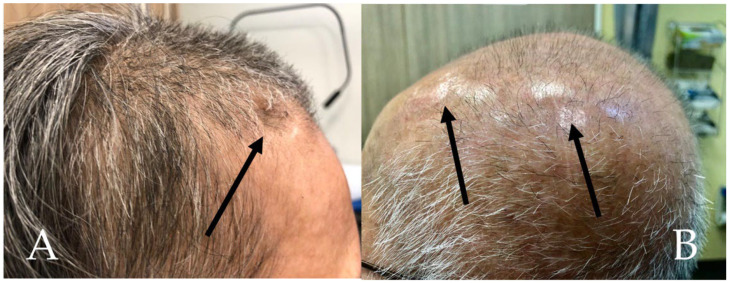
(**A**) A clinical photograph of the burrhole depression (black arrow) of a right frontal burrhole after a burrhole craniostomy where no burrhole cover was used. (**B**) A clinical photograph of a patient who received Design 2 in the left parietal burrhole (posterior arrow) and Design 1 in the frontal burrhole (anterior arrow), showing a good cosmetic outcome.

After burrhole craniostomy, patients experience variable degrees of wound healing, wound swelling reduction, and overlying soft tissue atrophy, resulting in postoperative scalp skin depressions [13]. While clinical outcomes of burrhole craniostomy are generally favorable, patients may often feel embarrassed and troubled from the residual scalp depression over the uncovered burrhole [20]. Im et al. reported that up to 64% of patients reported unacceptable cosmetic outcomes from uncovered burrhole craniostomy, resulting in functional handicaps in activities of daily living (ADLs) [13]. A clinical photograph to demonstrate a burrhole depression is depicted in Figure 2A, while in Figure 2B the craniostomy has been covered with the burrhole cover (Design 2). 

Despite the availability of burrhole covers, not all neurosurgeons place burrhole covers after craniotomies. A survey of neurosurgeons suggests that the reasons for this include a lack of proven benefit, technical difficulty, and fear of increased complications [21]. Given this hesitancy and the paucity of research investigating the utility of burrhole covers in clinical practice, we sought to evaluate Design 2 in three domains: cosmesis, quality of life, and clinical safety.

## 2. Materials and Methods

We conducted a cross-sectional study of consecutive patients who underwent burrhole drainage of chronic subdural hematoma with and without burrhole cover placement in the National University Hospital, Singapore from 1 April 2017 to 30 March 2021. Institutional review board approval was received for this study (domain-specific review board (DSRB) (National Healthcare Group) number: 2020-01458). Written informed consent was not required for electronic medical record collection, and verbal informed consent needed for interviews was obtained by the two investigators (E.M.S.T. and A.A.T.). The inclusion criteria included all patients who had undergone burrhole drainage for cSDH, and the exclusion criteria were patients who were under 21 years of age, underwent a mini-craniotomy as the initial operation for cSDH, or received burrhole craniostomy for other neurosurgical conditions. Patients were identified retrospectively from the Operative Theatre Records System by searching for patients who had undergone burrhole drainage, and then they were screened for eligibility by the two investigators (E.M.S.T. and A.A.T.). The independent variable was the use of a burrhole cover to cover burrhole craniostomy. Other dependent variables and potential confounders—including patient characteristics such as age and gender, comorbidities, use of antiplatelet and anticoagulant drugs, and follow-up time—were also collected. 

The three primary outcomes measured and collected were as follows:

(1) Cosmesis, as measured by Aesthetic Numeric Analog (ANA)—a patient-rated cosmetic satisfaction score.
(2) Quality of life, as measured by the EQ-5D-3L and the Visual Analog Scale (VAS).
(3) Safety, as measured primarily by rate of infection and cSDH recurrence. Secondary safety outcomes such as functional independence were also recorded.

### 2.1. Data Source and Collection

Electronic medical records from the primary institution were collected and reviewed by 2 independent investigators. The data collected included patient demographics such as age, gender, and race; comorbidities such as hypertension, hyperlipidemia, and diabetes mellitus; and current medications, such as antiplatelet or anticoagulant drugs; as well as operative procedures, postoperative course, follow-up history, complications, and relevant imaging findings, such as radiological depth of depression.

**Figure 3:**
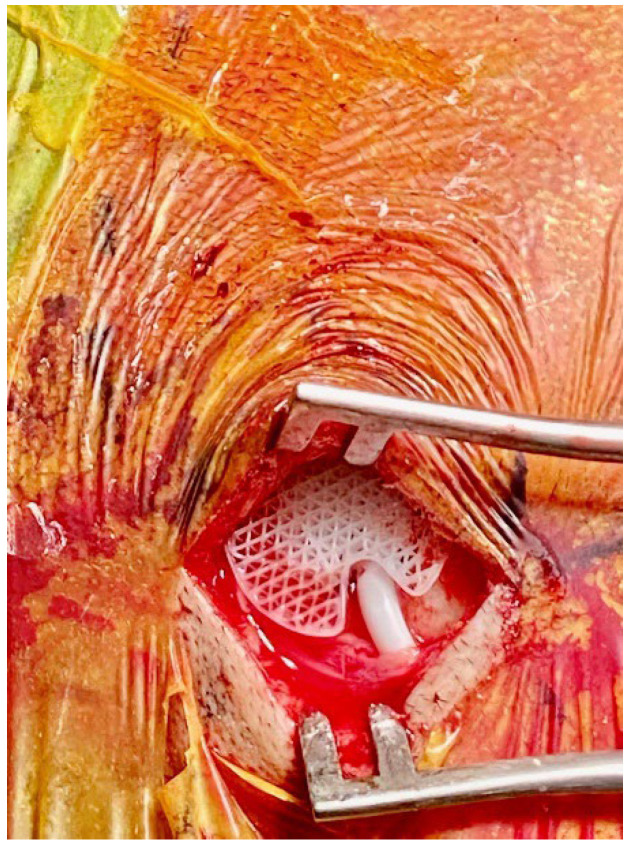
An intraoperative photograph of Design 2 inserted into a burrhole with a subdural drain visible.

The standardized operative technique for burrhole craniostomy was practiced in the institution. The number of burrholes (either 1 or 2) was decided based on the extent of the cSDH. The subdural hematoma was then flushed with gentamicin-impregnated Hartmann solution until clear returns were achieved. A subdural or extraosseous drain was then placed into either burrhole incision. Patients who received Design 2 all had about 2–3 cm of the distal tip of the drain placed in the subdural space under direct vision. The subdural drain used was the Medtronic standard barium-impregnated ventricular catheter (Medtronic). The Design 2 implant was then fitted snugly over the drain (Figure 3). This drain was tunneled through the skin away from the incision, anchored to the skin, and connected to a Becker^®^ External Drainage and Monitoring System (Medtronic). The external drainage system was positioned 20–30 cm below the external acoustic meatus to facilitate the drainage of subdural fluid. Of those who received Design 1 without Design 2, all received extraosseous drains. For those with extraosseous drains, Design 1 was first placed, before a Steril MVAC System drainage catheter, size 10 Fr (Steril Medical, Singapore), was connected to a passive drainage bottle without suction and placed at the bottom of the bed. For the remaining burrholes where a drain was not placed, they received either Design 1 or no burrhole cover. Postoperatively, patients were sent to the neurosurgical high-dependency unit and kept supine on bedrest for 24–48 h prior to being transferred to the general ward. Drains were kept in situ for 24–48 h following the operation, and a postoperative CT scan was obtained prior to drain removal.

**Figure 4:**
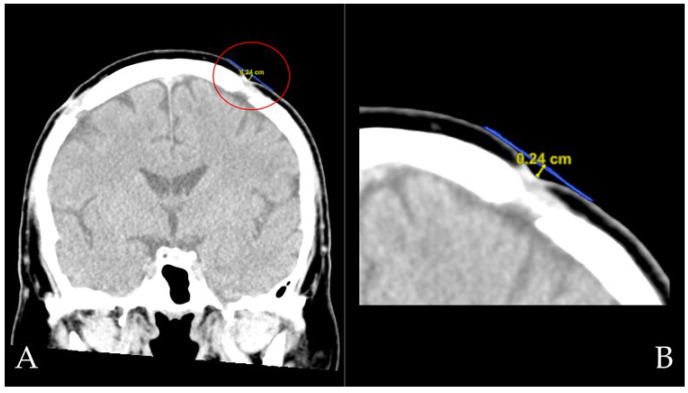
(**A**) A coronal CT image showing how radiological scalp depressions were measured, with an encircled scalp depression. (**B**) A magnified image of the scalp depression, to highlight how the distance was measured from the bottom of the depression to the hypothetical scalp line.

Medical records for both hospitalization and future follow-ups were recorded for clinical outcome measures, including recurrent cSDH, postoperative wound and central nervous system infections and drain-related complications (including iatrogenic acute subdural hemorrhage), and length of hospital stay. Patients who had bilateral cSDH burrhole craniotomies had two sets of data collected—one for each side. The definition of recurrence was in accordance with the Cambridge cSDH trial—a symptomatic, ipsilateral re-accumulation of cSDH seen on radiological imaging and requiring reoperation within 6 months of the index operation [22]. Radiological scalp depression was measured per burrhole. A radiological depression was noted if a scalp depression was seen on a coronal-view CT scan at least 2 months postoperatively. The depth of scalp depressions was measured from the patient’s most recent coronal brain CT if available. An increased contrast level was used to best visualize the surrounding scalp skin, and the depth of the depression was then measured as the distance from the hypothetical scalp line (extended from the adjacent normal scalp surfaces) to the deepest point of the depression (Figure 4). 

Telephone interviews consisted of questions from the Aesthetic Numeric Analog (ANA) Scale and the EQ-5D-3L. Assessment of the patient’s mRS at the time of the interview was performed using the simplified modified Rankin Scale Questionnaire [23]. Contact information of patients who received burrhole craniostomy was first identified from the original sample. All patients who were not labelled as deceased at the point of data collection were contacted via a letter of invitation, to which they could respond with a convenient interview timing. Patients were subsequently called by the two investigators (E.M.S.T. and A.A.T.) with a preapproved script and questionnaire. Interviews were conducted in a language suitable for the participant (English, Chinese, Malay, or Tamil), where validated translations from the EuroQol Group were used. For patients who were unable to answer the questionnaire at the point of the telephone interview, caregivers responded as proxies on the patients’ behalf, where validated proxy versions of the questionnaire were used. The EQ-5D-3L [24] is a widely used generic measure of health status consisting of two parts: The first part is the EQ-5D descriptive system, which assesses health in five dimensions (i.e., mobility, self-care, usual activities, pain/discomfort, and anxiety/depression), each of which has three levels of responses (i.e., no problems, some problems, or extreme problems/inability). These 5 descriptive dimensions were combined into an EQ-5D summary index for each patient, calculated using the EQ-5D-3L value set validated in Singapore by the time trade-off (TTO) method. [25] The second part of the EQ-5D questionnaire consists of a Visual Analog Scale (VAS), on which the patient rates their perceived health state from 0 (the worst imaginable health) to 100 (the best imaginable health). The ANA represents a patient’s satisfaction with the aesthetic results of the scar or depression created by burrhole craniostomy. The ANA was first used for evaluating the cosmetic outcomes of burrhole craniostomy by Vasella et al in 2018 [20], while it was first introduced to analyze cosmetic outcomes after maxillofacial and plastic surgery by Funk et al. [26] Patients ranked their satisfaction from 0 (not satisfied) to 10 (perfectly satisfied). This version and the appropriate translations used were approved by the DSRB and slightly modified from the original version by Funk et al, as demonstrated in Supplementary Materials. Patients also discussed in the interview whether their scalp depressions were visible to themselves, which was referred to as visible scalp depression. Across all study groups, the same sets of standardized interview scripts and questionnaires were used to minimize interviewer bias. Non-response bias was minimized by contacting each participant via letters of invitation, repeated attempts at calling (up to three times), and allowing participants to respond in various languages or via proxy if necessary. The number of cases of cSDH during the study period determined the sample size.

### 2.2. Statistical Analysis

All statistical analysis was performed using R version 4.0.2 (2020-06-22). The Shapiro–Wilk test was used to test variables for normality. Statistical analysis using the Mann–Whitney U test or the Kruskal-Wallis test was performed for non-normally distributed continuous variables, while the chi-squared test was used for categorical variables. An adjusted multivariate logistic regression model was also used to identify independent associations of burrhole cover use with aesthetic outcomes, which included confounding for age, gender, EQ-5D Index, and months from follow-up. Adjusted odds ratios were calculated and presented as an adjusted odds ratio (adj OR) with a 95% confidence interval (CI). Any *p*-values < 0.05 were considered statistically significant. Analyses of patient demographics, clinical outcomes (i.e., infections, functional outcomes, and hospital stay), and interview outcomes were performed per patient, but analyses of cSDH recurrence and drain complications were analyzed per burrhole surgery. Analysis of radiological depth of depression was measured per burrhole. Patients who had received at least one Design 2 were analyzed in the Design 2 group. There were minimal missing data (<12%), and any missing data were removed by complete-case analysis. Sensitivity and subgroup analyses were not conducted.

## 3. Results

### 3.1. Baseline Characteristics

Clinical and patient outcomes for a total of 126 patients who experienced cSDH and required burrhole craniostomy were analyzed in our study; 27 patients and 33 subdural hematomas received Design 2, 21 patients received only older versions of Design 1 without Design 2, and 78 patients received no burrhole cover at all. Baseline characteristics of the total number of patients and the number of interviewed patients across the three groups can be found in Table 2. The patients with no burrhole cover were found to be marginally older than the other groups (Table 2). 

A total of 109 patients who underwent burrhole surgery from 1 April 2017 to 30 March 2021 were contacted for interview; 13 of the 126 extracted patients were deceased at the point of interview, and 4 had since undergone mini-craniotomy for recurrent SDH after their initial operation, making them unsuitable for evaluation for the cosmetic appearance of the original burrhole-related skin depression. A total of 57 patients consented to being interviewed, and 52 declined or were uncontactable, with a response rate of 52.3%. There were no significant differences in the baseline characteristics of the interviewed group except for diabetes (Table 2). At the point of the call, 45 patients had an mRS of 0–2 and 12 had an mRS of 3–6, with the overall median [interquartile range] duration of follow-up being 24 [15, 37] months from the operation. Those with Design 2 had a shorter median follow-up time compared to the Design 1 and no burrhole cover groups (Table 2). This difference was anticipated, since Design 2 has been introduced more recently than Design 1 and its use has recently increased.

### 3.2. Cosmetic Outcomes

Table 3 summarizes the cosmetic outcome measures from radiological measurements and patient-reported outcomes from interviews. 

#### 3.2.1. Radiological Results: Depth of Scalp Depression

A total of 202 burrholes were included in the measurement of scalp depressions. Burrhole measurements were excluded from radiological depth measurement if there was no available postoperative CT brain scan at least 2 months after the operation. The median [interquartile range] time at which the depression depth was measured postoperatively was at 7.90 months [3.53, 15.36], and there were no significant differences in follow-up time or time from interview to data collection between the three groups. The depth of scalp depression was significantly less in Design 1 and 2 compared to the group with no burrhole cover (*p* < 0.001) (Table 3).

#### 3.2.2. Results from Patient Interviews: ANA and Visible Depression

**Figure 5:**
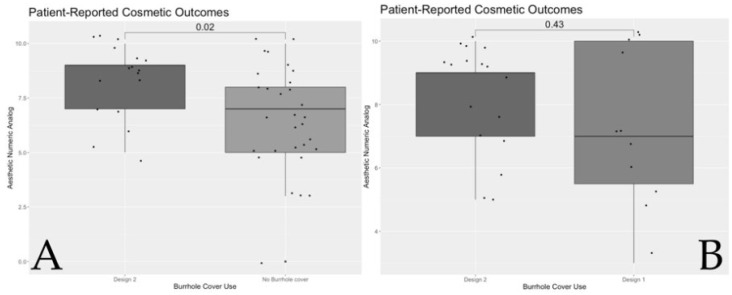
(**A**) Boxplot showing the differences in the median primary outcome measure—Aesthetic Numeric Analog—between those who had received Design 2 and no burrhole cover. (**B**) Boxplot showing a similar analysis among those who had received Design 2 and Design 1. Statistical analysis: Mann–Whitney U test; significance = *p* < 0.05.

In a three-way comparison between the three groups of patients, the ANA approached statistical significance (Table 3). Hence, further analysis was conducted to investigate two-way comparisons between the three groups. Overall patient satisfaction with Design 2 was significantly higher compared to no burrhole cover (*p* = 0.019) (Figure 5A). Cosmetic outcomes in Design 2 were not statistically significantly higher compared to Design 1 (*p* = 0.43) (Figure 5B). When patients were asked about their perceived visible scalp depressions, there were significantly fewer patients in Design 1 and 2 with visible scalp depressions compared to the group with no burrhole cover (*p* = 0.005) (Table 3). This corresponds with the radiological differences in depth of scalp depression.

The use of no burrhole cover was found to be predictive for having significantly lower ANA scores for cosmetic outcomes (defined as ANA score ≤ 7) upon multivariate analysis after adjustment for age, gender, and quality of life (EQ-5D Index), along with the number of months from follow-up (Table 4).

### 3.3. Quality-of-Life Outcomes

**Figure 6:**
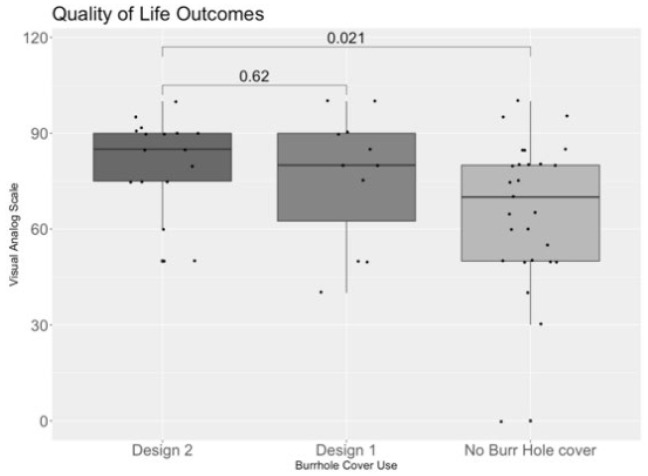
A boxplot comparing the Visual Analog Scale between those who had received Design 2, Design 1, and no Burrhole Cover. Statistical analysis: Mann–Whitney U test; significance = *p* < 0.05.

There were significant differences in the quality of life as measured by the EQ-5D Index among the three groups—likely lower in those with no burrhole cover than Design 1 or 2—and the VAS scores also trended towards significance when compared between the three groups (Table 5). Upon further analysis, VAS was significantly higher in those with Design 2 compared to those with no burrhole cover (*p* = 0.021), but was not statistically significant when compared to those with Design 1 (*p* = 0.62) (Figure 6).

### 3.4. Clinical Safety Outcomes 

There were no significant differences in the rate of cSDH recurrence between the three groups (*p* = 0.218). There were no other drain complications, such as iatrogenic acute SDH or drain malposition, in patients who received Design 2. There was one drain complication in the Design 1 group, where an extraosseous drain remained stuck to the burrhole cover after an unsuccessful postoperative drain removal. In this case, the affected burrhole cover and extraosseous drain were removed under local anesthesia, and the patient did not suffer any other drain-related adverse events, such as iatrogenic acute subdural hemorrhage or infection. At 3 months’ follow-up, the patient was well and there was no associated neurological decline. Overall, there were no significant differences in postoperative drain complications between the three groups of SDHs (*p* = 0.142).

Postoperative infections included wound and central nervous system infections. There were no significant differences in infection rates between the three groups (*p* = 0.721) (Table 6). The infection rate among those who had undergone burrhole craniostomy for cSDH was low, with only one patient becoming infected with an empyema among those without a burrhole cover. This patient presented with a 5-day history of headache and purulent discharge from the frontal and parietal burrhole wounds on postoperative day 20 after an uneventful discharge. He subsequently underwent a wound debridement and washout of burrhole incisions, where intraoperative cultures grew coagulase-negative *Staphylococcus* and methicillin-sensitive *Staphylococcus aureus* (MSSA). He was treated with intravenous cefazolin and was subsequently discharged well with oral clindamycin.

There were no significant differences in functional outcomes (*p* = 0.103), mortality (*p* = 0.734), or length of hospital stay between the three groups of patients (*p* = 0.660) (Table 6).

## 4. Discussion

Our findings indicate that patients with Design 2 had better cosmetic outcomes and quality of life than patients with no burrhole covers, with similar cosmetic and quality of life outcomes to the previously studied Design 1. Furthermore, Design 2 had good clinical safety outcomes and no association with increased SDH recurrence, infection, or drain-related complications.

Cosmetic outcomes in neurosurgery tend to be overlooked as an outcome measure, as they are not perceived to be immediately crucial to medical outcomes [27]. Multiple studies have investigated the cosmetic outcomes of patients after 3D reconstruction, typically for cranioplasty [28,29], and only two studies have analyzed the cosmetic outcomes after burrhole reconstruction with titanium burrhole covers. In other surgical disciplines, cosmetic outcomes are associated with quality of life, impaired social and emotional functioning, and depressive symptom scores [30]. Our results are consistent with the findings of previous studies that reported significantly higher patient satisfaction in those who received burrhole covers than in those who did not [13,20]. This could be attributed to objective and subjective scalp depression reduction. Similarly, the quality of life interview scores of patients with Design 2 were higher than those with no burrhole cover. In general, health-related quality of life (HRQoL) has not previously been well-reported in groups who underwent burrhole craniostomy. However, notably, Im et al reported an increase in quality-of-life outcomes after titanium burrhole reconstruction [13]. Hence, Design 2 may similarly improve quality-of-life outcomes by removing scalp depressions.

Our study also showed no significant differences in safety outcomes between the three groups. No association was observed between Design 2 use and cSDH recurrence or rates of infection and drain-related complications; this finding is comparable to our previously published studies on Design 1 [16], as well as similar studies on titanium burrhole covers [13,20]. This could encourage surgeons to use burrhole covers, given that increased complications were among the top five reasons cited for not using burrhole covers [21]. One complication was found in a Design 1 product, where the extraosseous drain became stuck to its superior surface. Despite extensive investigation by both surgeons and the manufacturers, no specific cause of this complication was identified.

Materials used for 3D-printed scaffolds must have several criteria for successful utilization, some of which include biocompatibility, biodegradability, strength and stiffness comparable to biological tissues [31] and, crucially, porosity. Biocompatibility refers to integrating well with the host material so as not to provoke an adverse immune response [32], while biodegradability is necessary for the scaffold to support tissue regrowth during its natural lifespan before being naturally resorbed into the body [33]. The rate of bioresorption of the scaffold in vivo should also be controlled and well-defined specifically to the host tissue [34]. Mechanical strength refers to the structural integrity of the scaffold to support the region at the scaffold–tissue interface and prevent collapse during daily activities, while not impeding bone formation [35,36]. Finally, scaffold microarchitecture and the size, distribution, and connection of pores affect patterns of bony ingrowth [37] and vessel development [38], and promote further tissue regrowth via cell entrapment [36]. It has been suggested that pore sizes greater than 250 μm allow for greater vessel ingrowth than smaller pores [39,40]. Significantly, an interconnected porous architecture leads to excellent tissue regeneration and vascularization [41], while pore pathways that are only partially connected inhibit cell migration [42]. This is closely linked to osseointegration properties, describe a material’s ability to encourage immature and pluripotent stem cells to differentiate into chondrocytes and osteoblasts, allowing for vascular integration [43]. Poor osseointegration could impair consequential bone repair and long-term implant functionality by decreasing mechanical stability [36]. Our PCL burrhole covers prove advantageous in these respects compared to existing popular alternatives, including the titanium burrhole covers whose cosmetic outcomes were previously studied [13,44], or porous polyethylene [8]. Other materials previously reported to cover burrhole defects include PMMA [9], HA [13], mineralized collagen [12], and autologous bone [6,7]. These materials are less ideal for use as burrhole covers, for reasons such as poor cost-effectiveness, time-consuming application, or the lack of interconnected pores, which are important for tissue integration [8,13]. In comparison, our study on Design 2 reaffirms the benefits of using PCL, which is highly biocompatible and biodegradable, has high biomechanical strength, and has a fixed interconnected pore microarchitecture that allows integration with osseous tissue [8,13]. 

With reference to biocompatibility, while titanium is biologically inert [45], PMMA may exhibit a toxic exothermic reaction, which could potentially be neurotoxic [14]. Meanwhile, PCL and PCL-incorporated tricalcium phosphate (PCL/TCP) have been shown to promote cell regeneration for traumatic brain injury and do not cause inflammation, emphasizing their safety to tissues [46,47]. Additionally, the biodegradation properties of PCL and PCL/TCP scaffolds have shown an appropriately lengthy degradation life, according to the initial molecular weight [48], allowing time for the replacement and healing of bone and vascular tissues over their functional lifespan before resorption [34]. The high biodegradability of PCL implants is achieved via hydrolytic and lipase-type enzymatic degradation [49]. This can be compared to permanent non-resorbable implants made of porous polyethylene, HA, and titanium [36,50], which could introduce additional concerns, including a theoretical delayed onset of infection, and some case reports of porous-polyethylene-related intense foreign body reaction have emerged [51,52]. Furthermore, the snap-fit design of the PCL burrhole covers does not require additional screws [15]. Titanium screws, in contrast, could theoretically result in screw displacement or scalp injury.

PCL has shown to be used under harsh mechanical, physical, and chemical conditions without significant loss of properties [53]. Similarly, PCL scaffolds have previously been shown to demonstrate biomechanical strength 60% that of normal bone in vivo [54]. Recent studies on PCL have demonstrated enhanced mechanical stability [55] in comparison to ceramic materials, which have high brittleness and high fatigue strength [36].

**Figure 7:**
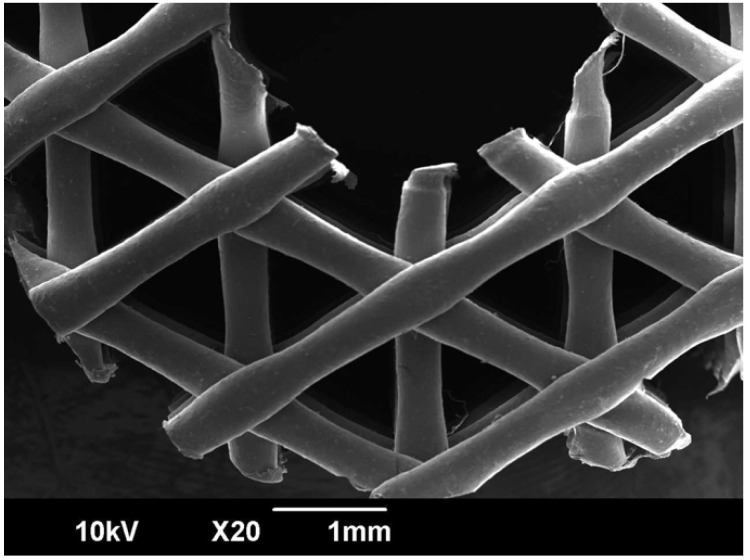
A scanning electron microscope image of Osteoplug^TM^—a PCL burrhole cover—demonstrating regular, highly interconnected porosity in the scaffold microarchitecture.

**Figure 8:**
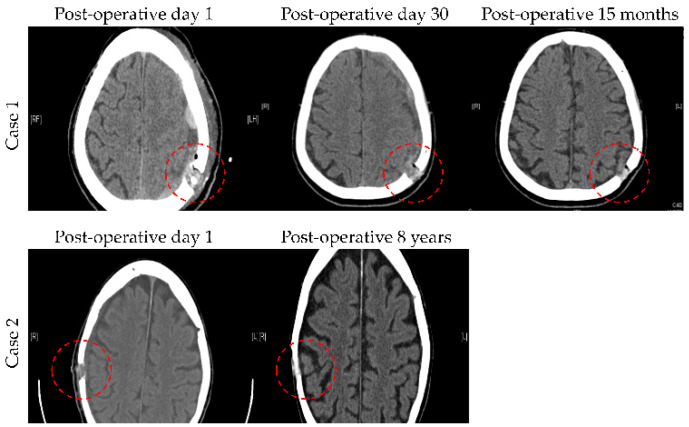
Two cases of serial postoperative computed tomography images demonstrating the bone ingrowth sufficiently covering the burrhole defect at varying stages of implantation over time. Reprinted/adapted with permission from Ref. [16]. 2020, Future Medicine, *Journal of 3D Printing in Medicine*.

Finally, PCL has a 400–600 μm pore size with a porosity of 60%–70% [17], as demonstrated by the scanning electron microscope image shown in Figure 7. This structured interconnected porosity allows PCL burrhole covers to achieve bony growth over time, as shown in Figure 8 [16]. Together with micro-computed tomography scans and histological staining, this provides evidence to confirm significant tissue infiltration, i.e., sufficient bone growth to cover the previous burrhole defect [16]. In comparison, titanium burrhole covers have single isolated pores, which could cause discontinuous patterns of bone ingrowth [37].

Our results add to the scarce body of literature to demonstrate how burrhole covers are safe and suitable for use in patients following evacuation of cSDH, achieving good cosmetic outcomes. As a pilot, this study also sets the stage for further research into the potential for the use of both PCL and Osteoplug^TM^-C (Design 2) on a larger scale. The limitations of our study include the retrospective nature of the electronic medical record collection, where potential confounders may not have been collected and adjusted for. Another limitation was the relatively low response rate among the participants approached for interview. The reasons stated for non-response mainly included time limitations, participants having moved to a nursing home, or communication difficulties. There were also several participants who were uncontactable. Patients may also not have been technologically adept or may have been wary of scam calls, which were prevalent over the interview period. However, our response rate was comparable to those of other studies analyzing quality-of-life data in Singapore [25]. Patients were not able to be interviewed at the same timepoint postoperatively, although this was accounted for in the multivariate analysis. Some patients underwent revision operations or were lost to follow-up, so postoperative radiological depth of burrhole depressions was unable to be measured. The Aesthetic Numeric Analog was also not a scale that had been officially validated in a Singaporean cohort in different languages, which could have limited the interpretability of the patient-reported outcomes.

As this was a pilot study with a relatively small cohort, further research with a larger population will be crucial for confirming the clinical and cosmetic outcomes of Design 2. Future work could also involve interviewing surgeons as to their preference and the ease of use of Design 1 to ascertain whether Design 2 is the preferred burrhole cover version among surgeons.

## 5. Conclusions

A redesign of a PCL burrhole cover for placement of subdural drains was observed to provide good cosmetic outcomes and comparable safety outcomes. Implant use was not associated with increased complications and showed the potential to reduce scalp-depression-related cosmetic handicaps in patients. Our pilot study indicates that the use of PCL burrhole covers provides good cosmetic benefits as compared to no burrhole cover use, but larger clinical studies will be necessary to validate this association and translate it into improved clinical outcomes. 

## Figures and Tables

**Table 1:** Latest designs of Osteoplug^TM^ and in-text references.

Commercial Name	Older Versions of Osteoplug^TM^	Osteoplug^TM^-C
In-Text Reference	Design 1	Design 2
Image Reference Examples	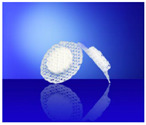 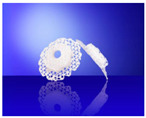	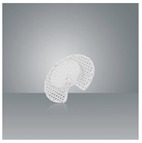

**Table 2:** Comparing the baseline characteristics of patients.

	Design 2	Design 1	No Burrhole Cover	*p*
Total Number of Patients	27	21	78	
Age (Median [IQR])	69 [62, 79.5]	70 [57, 75]	75 [69, 82.75]	0.023
Female (*n*, %)	9 (33.3)	6 (28.6)	21 (26.9)	0.817
Race (*n*, %)				0.586
Chinese	18 (66.7)	17 (81.0)	60 (76.9)	
Indian	1 (3.7)	1 (4.8)	3 (3.8)	
Malay	6 (22.2)	1 (4.8)	7 (9.0)	
Others	2 (7.4)	2 (9.5)	8 (10.3)	
Hypertension (*n*, %)	12 (44.4)	9 (42.9)	46 (59.0)	0.249
Hyperlipidemia (*n*, %)	13 (48.1)	7 (33.3)	46 (59.0)	0.100
Diabetes Mellitus (*n*, %)	7 (25.9)	2 (9.5)	28 (35.9)	0.057
Antiplatelet Drugs (*n*, %)	7 (25.9)	3 (14.3)	25 (32.1)	0.264
Anticoagulation (*n*, %)	1 (3.7)	2 (9.5)	5 (6.4)	0.714
Number of Patients Interviewed	17	11	29	
Age (Median [IQR])	68 [62, 76]	67 [48, 74.5]	71 [64, 83]	0.285
Female (*n*, %)	5 (29.4)	2 (18.2)	8 (27.6)	0.785
Race (*n*, %)				0.362
Chinese	14 (82.4)	9 (81.8)	22 (75.9)	
Malay	3 (17.6)	0 (0.0)	2 (6.9)	
Indian	0 (0.0)	1 (9.1)	1 (3.4)	
Others	0 (0.0)	1 (9.1)	4 (13.8)	
Hypertension (*n*, %)	8 (47.1)	4 (36.4)	16 (55.2)	0.557
Hyperlipidemia (*n*, %)	8 (47.1)	3 (27.3)	19 (65.5)	0.083
Diabetes Mellitus (*n*, %)	3 (17.6)	0 (0.0)	14 (48.3)	0.005
Poor Function (mRS 3-6) at Time of Interview (*n*, %)	2 (11.8)	1 (9.1)	9 (32.1)	0.146
Time from Operation to Interview (Months, Median [IQR])	15 [6, 29]	24 [15, 37]	26 [19, 39]	0.024

Abbreviations: *p*: *p*-value, cSDH: chronic subdural hemorrhage, IQR: interquartile range, mRS: modified Rankin Scale. Statistical analysis: Kruskal-Wallis test for continuous variables, chi-squared test for categorical variables; significance = *p* < 0.05.

**Table 3:** Cosmetic outcome measures.

	Design 2	Design 1	No Burrhole Cover	*p*
Number of Burrholes with Suitable Postoperative CT	17	43	142	
Radiologically Measured Depth of Depression (mm, Median [IQR])	0.00 [0.00, 1.30]	0.00 [0.00, 1.50]	2.50 [1.42, 3.50]	<0.001
Number of Patients Interviewed	17	11	29	
ANA (Median [IQR])	9 [7, 9]	7 [5.5, 10]	7 [5, 8]	0.076
Visible Scalp Depression (*n*, %)	2 (12.5)	2 (18.2)	16 (57.1)	0.005

Abbreviations: *p*: *p*-value, ANA: Aesthetic Numeric Analog, IQR: interquartile range. Statistical analysis: Kruskal-Wallis test for continuous variables, chi-squared test for categorical variables; significance = *p* < 0.05.

**Table 4:** Multivariate logistic regression for low Aesthetic Numeric Analog score.

	Adj OR	95% CI	*p*
Presence of Osteoplug^TM^			
Older Design 1 Used	4.16	0.73–28.52	0.119
No Burrhole Cover	4.55	1.09–22.68	0.047
Age	0.95	0.90–1.00	0.066
Gender	1.15	0.29–4.66	0.845
EQ-5D Index	0.41	0.11–1.34	0.154
Months from Follow-Up	0.99	0.95–1.04	0.759

Abbreviations: Adj OR: adjusted odds ratio, CI: confidence interval, *p*: *p*-value, EQ-5D Index: EQ-5D Index Health State values.

**Table 5:** Quality-of-life outcome measures.

	Design 2	Design 1	No Burrhole Cover	*p*
Number of Patients Interviewed	17	11	29	
EQ-5D Index (Median [IQR])	1.00 [1.00, 1.00]	1.00 [0.93, 1.00]	0.72 [0.04, 1.00]	0.027
VAS (Median [IQR])	85 [75, 90]	80 [62.5, 90]	70 [50, 80]	0.059

Abbreviations: *p*: *p*-value, EQ-5D Index: EQ-5D Index Health State values, VAS: Visual Analog Scale from the EQ-5D-3L. Statistical analysis: Kruskal-Wallis test for continuous variables, chi-squared test for categorical variables; significance = *p* < 0.05.

**Table 6:** Clinical safety outcome measures.

	Design 2	Design 1	No Burrhole Cover	*p*
Number of Burrhole Surgeries	33	35	105	
cSDH Recurrence (*n*, %)	7 (23.3)	3 (8.6)	14 (13.3)	0.218
Drain Complications (*n*, %)	0 (0.0)	1 * (2.9)	0 (0.0)	0.142
Number of Patients	27	21	78	
Postoperative Infection (*n*, %)	0 (0.0)	0 (0.0)	1 ^^^ (1.4)	0.721
Poor 90-Day Functional Outcome (mRS 3-6) (*n*, %)	4 (17.4)	0 (0.0)	15 (21.1)	0.103
30-Day Mortality (*n*, %)	0 (0.0)	0 (0.0)	1 (1.3)	0.734
Duration of Hospital Stay (Median [IQR])	6 [3, 12]	8 [5, 10]	7 [4, 13]	0.660

* One drain complication occurred where the drain was stuck to the Design 1 burrhole cover. ^ One postoperative subdural empyema (methicillin-sensitive *Staphylococcus aureus* and coagulase-negative *Staphylococcus*) occurred in the group without burrhole covers. Abbreviations: n: number, N: total, *p*: *p*-value of Pearson’s chi-squared test between the listed variables, EQ-5D Index: EQ-5D Index Health State values, VAS: Visual Analog Scale from the EQ-5D-3L, ANA: Aesthetic Numeric Analog, IQR: interquartile range. Statistical analysis: Kruskal-Wallis test for continuous variables, chi-squared test for categorical variables; significance = *p* < 0.05.

## Data Availability

The data presented in this study are available upon request from the corresponding author. The data are not publicly available due to ethical restrictions.

## Supplementary Materials

The following supporting information can be downloaded at: www.mdpi.com/xxx/s1, Supplementary Materials: Questionnaire—Aesthetic Numeric Analog scale and visible scalp depressions.
