# Supplementary material for: Clinical Outcomes of 3D-Printed Bioresorbable Scaffolds for Bone Tissue Engineering—A Pilot Study on 126 Patients for Burrhole Covers in Subdural Hematoma
Source: Biomedicines. 2022 Oct 26;10(11):0. doi: 10.3390/biomedicines10112702 (PMC9687313; doi:10.3390/biomedicines10112702)
Supplement: Supplementary file 1 [file biomedicines-10-02702-s001.zip › biomedicines-1939695-supplementary-final.pdf]

biomedicines

MDPI

Supplementary

Questionnaire - Aesthetic Numeric Analog scale and Visible Scalp Depressions

Aesthetic Numeric Analog: Please select ONE value that describes how you feel about the aesthetic outcome (the cosmetic look) of your surgery?

0	1	2	3	4	5	6	7	8	9	10
Not satisfied at all										Perfectly satisfied

Note: adapted from Vasella F, Akeret K, Smoll NR, et al. Improving the aesthetic outcome with burr hole cover placement in chronic subdural hematoma evacuation-a retrospective pilot study. Acta Neurochir (Wien). 2018;160(11):2129-2135. doi:10.1007/s00701-018-3659-9 and Funk W, Podmelle F, Guiol C, Metelmann HR. Aesthetic satisfaction scoring - introducing an aesthetic numeric analogue scale (ANA-scale). J Craniomaxillofac Surg. 2012;40(5):439-442. doi:10.1016/j.jcms.2011.07.018

Visible Scalp Depressions

Interviewer: Do you still have a visible scalp depression at the site of the surgery?

Note that the EQ-5D and VAS is not included in this Appendix as it is not to be reproduced without permission from the EQ-5D Office.
